# Mammal defaunation leads to biotic homogenization of plant communities in tropical rainforests

**DOI:** 10.1002/ecy.70341

**Published:** 2026-03-16

**Authors:** Luiz Guilherme dos Santos Ribas, Nacho Villar, Valesca Zipparro, Sérgio Nazareth, Yuri Souza, Carlos Rodrigo Brocardo, Gabriela Schmaedecke, Luana Hortenci, Rafael Souza Cruz Alves, Mauro Galetti

**Affiliations:** ^1^ Centro de Pesquisa em Biodiversidade e Mudanças no Clima (CBioClima), Instituto de Biociências, Laboratório de Biologia da Conservação São Paulo State University (UNESP) Rio Claro Brazil; ^2^ Grupo de Pesquisa em Recursos Pesqueiros e Limnologia (GERPEL), Laboratório de Ictiologia e Estatística Pesqueira (ICTES), State University of Wester Paraná Toledo Brazil; ^3^ Department of Aquatic Ecology Netherlands Institute of Ecology NIOO‐KNAW Wageningen The Netherlands; ^4^ Lowland Tapir Conservation Initiative Institute for Ecological Research Campo Grande Brazil; ^5^ Universidade Federal da Fronteira Sul (UFFS), Campus Realeza Realeza Brazil; ^6^ Centro Nacional de Pesquisa e Conservação de Mamíferos Carnívoros (CENAP) Atibaia Brazil; ^7^ Traffic International David Attenborough Building Cambridge UK; ^8^ Centro de Pesquisa em Biodiversidade e Mudanças no Clima (CBioClima), Instituto de Biociências, Laboratório de Ecologia Espacial e Conservação (LEEC) São Paulo State University (UNESP) Rio Claro Brazil

**Keywords:** Atlantic forest, biotic homogenization, Brazil, *Euterpe edulis*, herbivores, top‐down control

## Abstract

Biotic homogenization is the process in which species communities become increasingly similar across different regions over time. This phenomenon has substantial ecological, evolutionary, and economic implications, primarily driven by human activities such as habitat destruction, invasive species introduction, and climate change. An underexplored driver of biotic homogenization is defaunation, particularly the loss or population decline of large herbivorous mammals and its consequences on plant communities. In this study, we examined how defaunation of medium‐ to large‐sized mammals, such as tapirs and peccaries, affects taxonomic biotic homogenization in seedling and sapling communities in tropical rainforests of South America. Using data from a 13‐year mammal‐exclosure experiment across four forest sites in the Brazilian Atlantic Forest, we investigated the effects of defaunation on both alpha and beta diversity to understand how it might contribute to biotic homogenization. Our results indicate that defaunation significantly increased alpha diversity in exclusion plots over time, contrary to expectations, with more pronounced effects at forest sites hosting more complete mammal assemblies, that is, with greater mammal abundance and diversity. In contrast, beta diversity decreased as exclusion treatments led to more spatially homogeneous plant communities, particularly at the site where exclusion treatment prevents access to the plant community by the most complete mammal assembly. This homogenization was driven by reduced species turnover and the dominance of a few plant species that thrive in the absence of mammal herbivores, including a palm *Euterpe edulis*, a bamboo *Merostachys neesii*, and a fern *Polybotrya cylindrica*. These findings suggest that the removal of medium‐ to large‐sized mammal herbivores can lead to both increased local species richness and decreased spatial heterogeneity, reshaping plant community structure across tropical forest landscapes. Our study highlights the critical role of large‐bodied herbivores in maintaining biodiversity at multiple scales and underscores the ecological consequences of their functional loss. This work provides essential insights for conservation efforts aimed at mitigating the impacts of defaunation and preserving the resilience of tropical forest ecosystems, positioning defaunation as a significant anthropogenic driver of biotic homogenization.

## INTRODUCTION

Biotic homogenization refers to the directional shift in beta diversity in which communities sharing the same species pool become increasingly similar over time in different areas (Olden & Rooney, 2006; Rolls et al., 2023). This process often leads to biodiversity loss, which has significant ecological, evolutionary, and economic costs (Olden et al., 2004). Specifically, biotic homogenization can affect community and ecosystem functioning, reduce stability, and weaken resistance to environmental change, ultimately undermining the resilience of ecosystems to future disturbances (Olden et al., 2004; Olden & Rooney, 2006; Rolls et al., 2023). The primary drivers of biotic homogenization are human activities, including direct impacts from habitat destruction, overharvesting, and the introduction of invasive species, as well as indirect impacts such as the cascading effects of climate change and pollution (Rolls et al., 2023). Therefore, addressing the causes and consequences of biotic homogenization is critical not only for understanding its occurrence and prevalence but also for developing effective strategies to mitigate its impacts and preserve biodiversity.

Among the many human‐driven factors contributing to biotic homogenization, the role of defaunation remains insufficiently understood. Defaunation—the human‐induced decline and extinction of wild animal populations and species—has become a widespread outcome of anthropogenic activities across the globe (Benítez‐López et al., 2019; Dirzo et al., 2014). Defaunation affects certain biological groups and locations more severely than others, especially the large‐bodied species in tropical environments (Cardillo et al., 2008; Young et al., 2016). The consequences of defaunation for ecosystems are profound, including direct effects such as the depletion of some ecosystem services provided by mammals (Bogoni et al., 2020; Galetti et al., 2021; Malhi et al., 2016; Pringle et al., 2023) and indirect effects such as the expected reduction in future tropical forest biomass, nutrient cycling, and carbon sequestration in many ecosystems (Brodie et al., 2025; de Paula Mateus et al., 2018; Schmitz et al., 2018). Despite these broad impacts on natural ecosystems across multiple ecological and evolutionary scales (Dirzo et al., 2014; Kurten, 2013; Young et al., 2016), a thorough understanding of how defaunation contributes to biotic homogenization remains unclear. For example, in a recent review by Rolls et al. (2023), no specific studies focused specifically on defaunation as a cause of biotic homogenization, despite its common attribution to factors such as biological invasions, changes in environmental conditions, and other anthropogenic disturbances.

In tropical forests, where large mammals are heavily impacted by poaching and habitat loss (Galetti et al., 2017, 2021), the loss of herbivory as a disturbance can substantially alter plant community structure (Jia et al., 2018). Defaunation disrupts selective feeding and reduces spatial heterogeneity, potentially leading to changes in the composition and diversity of plant communities. For example, studies in grassland ecosystems show that the experimental exclusion of large herbivores often results in reduced plant alpha diversity. This decrease in alpha diversity is typically associated with the increased dominance of a few species that thrive in the absence of large herbivores (Koerner et al., 2018). Experimental studies have shown that the removal of herbivores is associated with the homogenization of plant communities (Abella et al., 2019; Milligan et al., 2016; Speed et al., 2013; Watts et al., 2019). Yet evidence for this comes primarily from studies investigating trophic interactions between plants and herbivores in non‐natural ecosystems, such as livestock grazing.

Shifts in alpha diversity due to herbivory can have direct effects on beta diversity, especially when reductions in herbivory reduce the replacement of individuals within communities, since this might lead to more homogeneous species composition across sites. Large wild herbivores play a critical role in promoting ecological heterogeneity by introducing diverse disturbance patterns through their selective feeding and movement, which fosters spatial variability in plant communities (Hobbs, 1996; Milchunas & Lauenroth, 1993) and redistributes nutrient availability (le Roux et al., 2018, 2020; Villar et al., 2021). However, the defaunation of these herbivores reduces this source of heterogeneity, resulting in more uniform conditions across areas. Consequently, this reduction in disturbance variability may increase the spatial synchronization and similarity of plant species composition across areas over time, the phenomenon known as taxonomic biotic homogenization (Heino et al., 2015; Warren et al., 2007). In the absence of large wild herbivores, plant communities across landscapes might become more similar, with competition and abiotic factors becoming the primary drivers of species sorting, further promoting homogenization (Birtel & Matthews, 2016; Ohashi & Hoshino, 2014; Perea et al., 2014; Püttker et al., 2015). This process not only affects community composition but might also impact ecosystem resilience (Wang & Loreau, 2016), highlighting the importance of understanding these dynamics for effective conservation strategies (Baiser et al., 2012; Newbold et al., 2016; Olden et al., 2004). Therefore, understanding the relationship between alpha and beta diversity is crucial for comprehending the broader impacts of defaunation on biological communities, underscoring the importance of considering both levels of diversity in studies of biotic homogenization (Chase & Myers, 2011; Socolar et al., 2016).

Recent empirical evidence from mammalian herbivores‐exclusion studies in the Atlantic Forest of Brazil suggests that defaunation of wild herbivores might have an impact on biotic homogenization in forest ecosystems. Results from a 7‐year dataset indicate that defaunation of large herbivores reduces plant compositional beta diversity without major changes in alpha diversity (Villar & Medici, 2021). In addition, results from another similar exclusion experiment in a different region of the Atlantic Forest suggest that large herbivores buffer against global change by slowing down directional changes in plant community composition (Villar et al., 2020). Therefore, these two studies suggest that large herbivore defaunation may accelerate biotic homogenization in tropical forests.

**FIGURE 1:**
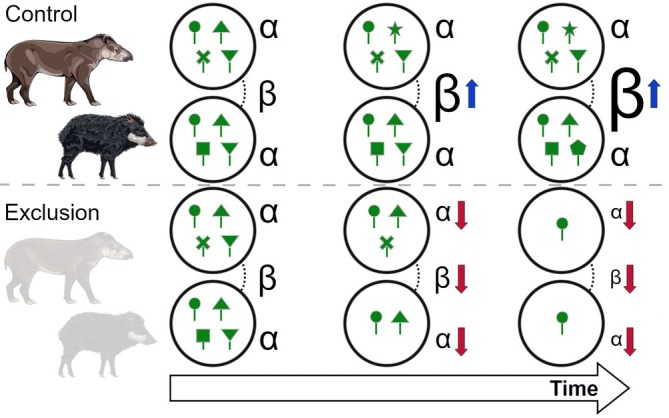
Scheme representing the expected changes in alpha (α) and beta (β) diversity in the experiment, with the upper section showing expectations for control plots and the lower section for mammal exclusion plots. Peccary and tapir illustrations by Fernanda Abra.

Here, we investigated how defaunation of medium‐ to large‐bodied mammals (i.e., >4 kg, such as agoutis, pacas, tapirs, deers, and peccaries) might lead to taxonomic biotic homogenization of seedling and sapling communities in tropical rainforests. Using data from a long‐term (13 years) mammal‐exclosure experiment in the Brazilian Atlantic Forest (The DEFAU‐Biota project; Brocardo et al., 2013; Emer et al., 2024; Souza et al., 2022; Villar et al., 2020), we examined the effects of the exclusion of medium‐ and large‐bodied terrestrial mammals on both alpha and beta diversity across four distinct protected forests of the Atlantic Forest of Brazil. Each forest features specific species composition and abundance of herbivorous mammals and plants. This setup allows us to experimentally explore how different levels of experimental defaunation might influence plant species diversity, composition, and taxonomic biotic homogenization, and whether these effects are consistent across ecological contexts within the same biome. We began by investigating alpha diversity to understand how defaunation influences local species richness and species dominance at each individual forest. We hypothesize that alpha diversity will decrease in exclusion plots, with more pronounced effects (i.e., larger differences between control and exclusion treatments) in forest sites where exclusion treatment prevents access by more mammals compared to control treatment (Figure 1). This expectation is based on the role of herbivores in promoting local diversity by acting as primary ecological disturbances that reduce the community share of dominant plant species through selective herbivory (Terborgh, 2012), as it is often the case in grassland ecosystems (Frank, 2005; Olff & Ritchie, 1998). Next, we analyzed how the variance in local community composition changes because of medium‐ to large‐sized mammal exclusion over time. Our primary hypothesis is that experimental exclusion will lead to increased taxonomic biotic homogenization across space, characterized by reduced beta diversity among exclusion treatment plots compared control treatment plots, in which we expect an increase in beta diversity (Figure 1). We expect the reduction in plant species diversity in exclusion plots will release the dominance of certain species across many plots within the same forest over time, leading to more similar plant communities. In addition, we hypothesize that this effect will be more pronounced in forests where exclusion treatment removed more mammals compared to control treatment. Therefore, by investigating both alpha and beta diversity along treatments and considering our defaunation gradient, we aim to provide a more comprehensive understanding of how defaunation influences the assembly of plant communities.

## METHODS

### Forest sites

The study was conducted in four continuous forest sites within protected areas of the Atlantic Forest in São Paulo State, Brazil: (1) Carlos Botelho State Park (CBO), located in São Miguel Arcanjo; (2) Itamambuca (ITA) and (3) Vargem Grande (VG), both located in the Núcleo Santa Virgínia sector of the Serra do Mar State Park, in São Luiz do Paraitinga and Natividade da Serra, respectively; and (4) Ilha do Cardoso State Park (CAR), located in Cananéia (Appendix S1: Figure S1). These areas were selected based on prior evidence of their importance for mammal conservation (Galetti et al., 2009).

Although these sites once shared similar mammal assemblages, they now differ due to varying histories of illegal hunting, which has led to the local extinction or population depletion of white‐lipped peccaries, tapirs, or both in some areas (Galetti et al., 2017). These differing hunting pressures have also resulted in variation in mammal abundance across sites (Appendix S1: Table S1), creating a natural experimental gradient useful for assessing the ecological consequences of defaunation.

Currently, ITA harbors both white‐lipped peccaries (*Tayassu pecari*) and tapirs (*Tapirus terrestris*), representing the most complete mammal assemblage among the sites, that is, with the greatest mammal abundance and diversity (Galetti et al., 2017). In contrast, both species are locally extinct (white‐lipped peccaries) or extremely rare (tapirs) in VG. In CAR, only white‐lipped peccaries are present and tapirs were extinct in the 1960s, while in CBO, during our first years only tapirs occur, and white‐lipped peccaries are absent or extremely rare (Appendix S1: Table S1). White‐lipped peccaries recolonized CBO and have increased their abundance only recently.

### Experimental design

We established 15 pairs of plots (3 × 5 m) in each forest site, replicating a total exclusion treatment for medium‐to‐large mammals along a paired control treatment (Appendix S1: Figure S1; Galetti et al., 2017; Villar et al., 2020). Plots were established in July 2009 in CBO and in July 2010 in CAR, ITA, and VG. Each pair of plots included an exclusion or closed plot (hereafter, exclusion treatment), which simulated the absence of medium‐to‐large non‐flying terrestrial animals, especially mammals, and a control or open plot (hereafter, control treatment), where all animals present in the experimental area had unrestricted access. The exclusion plots were enclosed by a 1.50‐m high fence, allowing access only to small rodents, small marsupials, birds, and invertebrates. The control plots remained open, with their boundaries delimited by polyvinyl chloride sticks (see Villar et al., 2020 for more details on the experimental design). Each pair of exclusion and control plots was separated from one other by 5–10 m to ensure environmental homogeneity, and there was a minimum distance of 200 m between pairs of replicates within the same forest site. Sunlight exposure and other abiotic conditions were similar between treatments within each pair of plots. Therefore, among forest sites, we have an experimental defaunation gradient. ITA shows more pronounced differences between exclusion and control plots due to its higher presence and abundance of medium‐ to large‐sized mammals. This is followed by CAR and CBO, with VG showing the least difference between treatments. See Appendix S1: Figure S1 for a visual overview of the experimental design.

Each experimental plot was subdivided into eight subplots of 1 m^2^ each, and in three of these subplots (Appendix S1: Figure S1), randomly selected, all seedlings and saplings between 10 cm and 1 m in height were labeled, measured, and identified to the highest taxonomic resolution possible, or morphotyped if taxonomic classification was inconclusive. Surveys were conducted every 6 months from July 2010 to 2018 and annually in 2019 and 2023. The window between 2019 and 2023 was due to restrictions given the COVID‐19 pandemic. During each survey, all newly identified individuals were included. Subsequently, seedling and sapling composition data were organized for each of the 18 sampling months to ensure consistency of identification across sampling periods and experimental areas, except for CBO, which does not have data for months 54, 60, 66, and 156.

### Data analysis

Data used in the analyses correspond to the surveys described in the section above, spanning 13 years of seedling and sapling community data for each forest site. We began by examining the responses of alpha diversity to experimental exclusion over time. For each forest site (i.e., ITA, CAR, CBO, and VG) and sampling occasion, we calculated seedling and sapling species richness per plot. We then fitted a linear mixed‐effects model to assess whether the interaction between sampling month and treatment, treated as fixed effects, explained differences in richness. Paired effects were controlled by incorporating treatment pairs as random effects in the model. The control treatment was used as the baseline intercept. A similar modeling procedure was applied to investigate the interaction between treatment and sampling month for other alpha diversity metrics: the Shannon–Weaver index (hereafter Shannon) and the inverse Simpson index (hereafter inverse Simpson). All alpha diversity analyses were performed using the diversity function from the vegan package (Oksanen et al., 2024) in the R environment (R Core Team, 2023).

Subsequently, for each forest site, each sampling occasion, and each treatment level (control vs. exclusion), we quantified abundance‐based multiple‐site dissimilarity among all plots. For each site, we calculated abundance‐based Bray–Curtis multiple‐site beta diversity considering each treatment and sampling period (Baselga, 2016). This approach follows the formulation of multiple‐site dissimilarity proposed by Diserud and Ødegaard (2007) and further developed by Baselga (2016), which provides a coherent measure of community differentiation among more than two plots. Thus, for each forest site and each sampling month, we obtained one multiple‐site beta diversity value per treatment, summarizing the compositional differentiation among all plots within that treatment at that time.

In addition to overall beta diversity, we partitioned abundance‐based dissimilarity into two additive components: (1) balanced variation in species abundance and (2) abundance gradients, following Baselga (2013) and Legendre (2014). These components are analogous to the turnover and nestedness components of dissimilarity typically used with presence–absence data (Baselga, 2010, 2012). Balanced variation reflects changes in species dominance among communities (i.e., shifts in the relative contribution of different species), whereas abundance gradients reflect consistent increases or decreases in the total abundance of all species at one site relative to another, without necessarily changing the dominance structure of the community.

To quantify uncertainty around these multiple‐site beta diversity estimates, we implemented a leave‐one‐out jackknife procedure at the plot level: for each site and the combination of treatments and sampling times, we recalculated total beta diversity, balanced variation and abundance gradients after sequentially removing each plot, generated jackknife pseudo‐values, and then obtained standard errors and approximate 95% CIs from the jackknife variance (Fortin et al., 2020). Finally, for each forest site, we modeled temporal trends in total beta diversity, balanced variation, and abundance gradients using linear models with sampling month (treated as a continuous covariate), treatment, and their interaction as fixed effects. In contrast to the alpha‐diversity analyses, these models were fitted to treatment‐level multiple‐site summaries (one value per treatment and time), and therefore did not include plot pair as a random effect. Balanced variation and abundance gradients were interpreted only when overall beta diversity exhibited a significant treatment‐by‐time interaction. All corresponding results are reported in Appendix S2.

All diversity metrics were computed using the decostand and vegdist functions from the vegan package (Oksanen et al., 2024), and the beta.multi.abund function from the betapart package (Baselga & Orme, 2012), implemented in the R environment (R Core Team, 2023). Prior to calculating each alpha and beta diversity metric, we applied the Hellinger transformation to the plant species composition data at each sampling period. This transformation downweights the influence of highly abundant species and ensures compatibility of beta diversity estimates with linear models (Legendre & Gallagher, 2001).

All linear models applied to alpha diversity indices, spatial beta diversity, and its components met the assumptions of linearity, independence of errors, homoskedasticity, normality of residuals, and absence of collinearity. These assumptions were verified by plotting residuals against fitted values and each model covariate. We also assessed residuals for potential temporal and spatial dependency. All models followed the general structure:

$$\text{Response}\;{\text{variable}}_{\mathrm{ij}}˜N\left(\mu_{\mathrm{ij}},\sigma^{2}\right);$$

$$\mu_{\mathrm{ij}}=\beta_{0}+\beta_{1}\times {\text{Time}}_{\mathrm{ij}}+\beta_{2}\times {\text{Treatment}}_{\mathrm{ij}}+\beta_{3}\times \left({\text{Time}}_{\mathrm{ij}}\times {\text{Treatment}}_{\mathrm{ij}}\right)+b_{i};$$

$$b_{i}˜N\left(0,\sigma_{\text{Pairs}}^{2}\right).$$

The random effect (*b*_*i*_) was included only for alpha diversity.

Finally, we used the interaction term between treatment and time from each linear model as an effect size to evaluate whether the impacts of mammal exclusion varied across forest sites according to the defaunation gradient. For each site and biodiversity metric, we extracted the interaction estimate and its 95% CI. We then performed pairwise comparisons of effect sizes across sites (ITA, CAR, CBO, and VG) by calculating the difference in estimates and the corresponding standard error, derived from the reported CIs. A *z*‐test was applied to assess statistical significance, and results were visualized in forest plots showing pairwise differences and their 95% CIs. This procedure was conducted in R (R Core Team, 2023). All analysis scripts, data, and supplementary model outputs are publicly available in Zenodo at https://doi.org/10.5281/zenodo.18261121 (Ribas et al., 2026). Additional details on the main effects of treatments and sampling months for alpha and beta diversity are also provided in the same repository.

## RESULTS

### Alpha diversity

**FIGURE 2:**
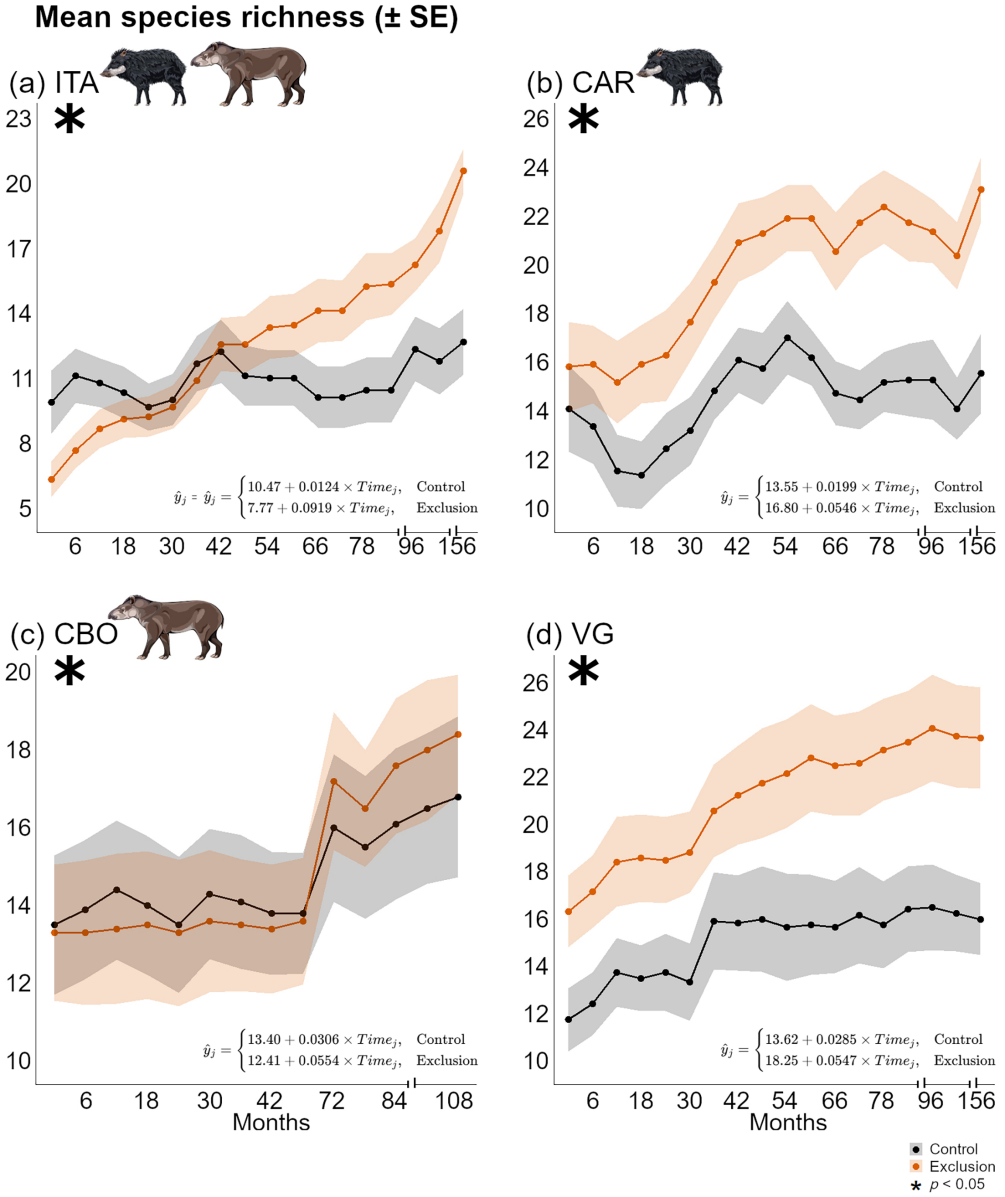
Temporal trends in species richness under control and exclusion treatments across four forest sites, based on linear mixed‐effects models. Each panel represents one site: (a) Itamambuca (ITA), (b) Ilha do Cardoso (CAR), (c) Carlos Botelho (CBO), and (d) Vargem Grande (VG). Lines represent mean species richness over time (± standard error). Linear equations represent temporal trends for each treatment, with the upper equation referring to control plots and the lower to exclusion plots. The notation *i* indicates each evaluated sampling period. Asterisks indicate statistically significant interactions (*p* < 0.05) between time and exclusion treatment, reflecting different rates of change between treatments. Tick intervals in horizontal axis represent changes in sampling constancy. Peccary and tapir illustrations by Fernanda Abra.

The number of plant species recorded in each forest site varied: ITA, characterized by the presence of white‐lipped peccaries and tapirs, had 170 seedling and sapling taxa over the study period; CAR, with the presence of white‐lipped peccaries, had 183 taxa; CBO, with the presence of tapirs, had 186 taxa; and VG, where large mammals are depleted, had 229 taxa. At the final sampling periods, exclusion plots were particularly dominated by three species: the palm *Euterpe edulis* (Arecaceae), the bamboo *Merostachys neesii* (Poaceae), and the fern *Polybotrya cylindrica* (Dryopteridaceae). Initially, ITA exhibited lower seedling and sapling richness under the exclusion treatment compared to the control, while the opposite was found for CAR and VG (Figure 2). This emphasizes the importance of considering the initial “pre‐experimental conditions” (*t* = 0) in all analyses and emphasizing changes in the trajectory (treatment‐by‐time interaction) as the process informing about the temporal effects of exclusion over time. Over the course of the study, species richness generally increased rapidly in exclusion plots across all sites, in which the rate of increase in exclusion treatment were higher than in controls (0.0919 in ITA, 0.0546 in CAR, 0.0554 in CBO, and 0.0547 in VG; equations in Figure 2). In VG, the most defaunated site, differences in richness between control and exclusion treatments were already present at the beginning; however, the significant time and treatment interaction indicates that these differences changed through time, with richness increasing more rapidly inside exclusions than in controls (Figure 2d).

**FIGURE 3:**
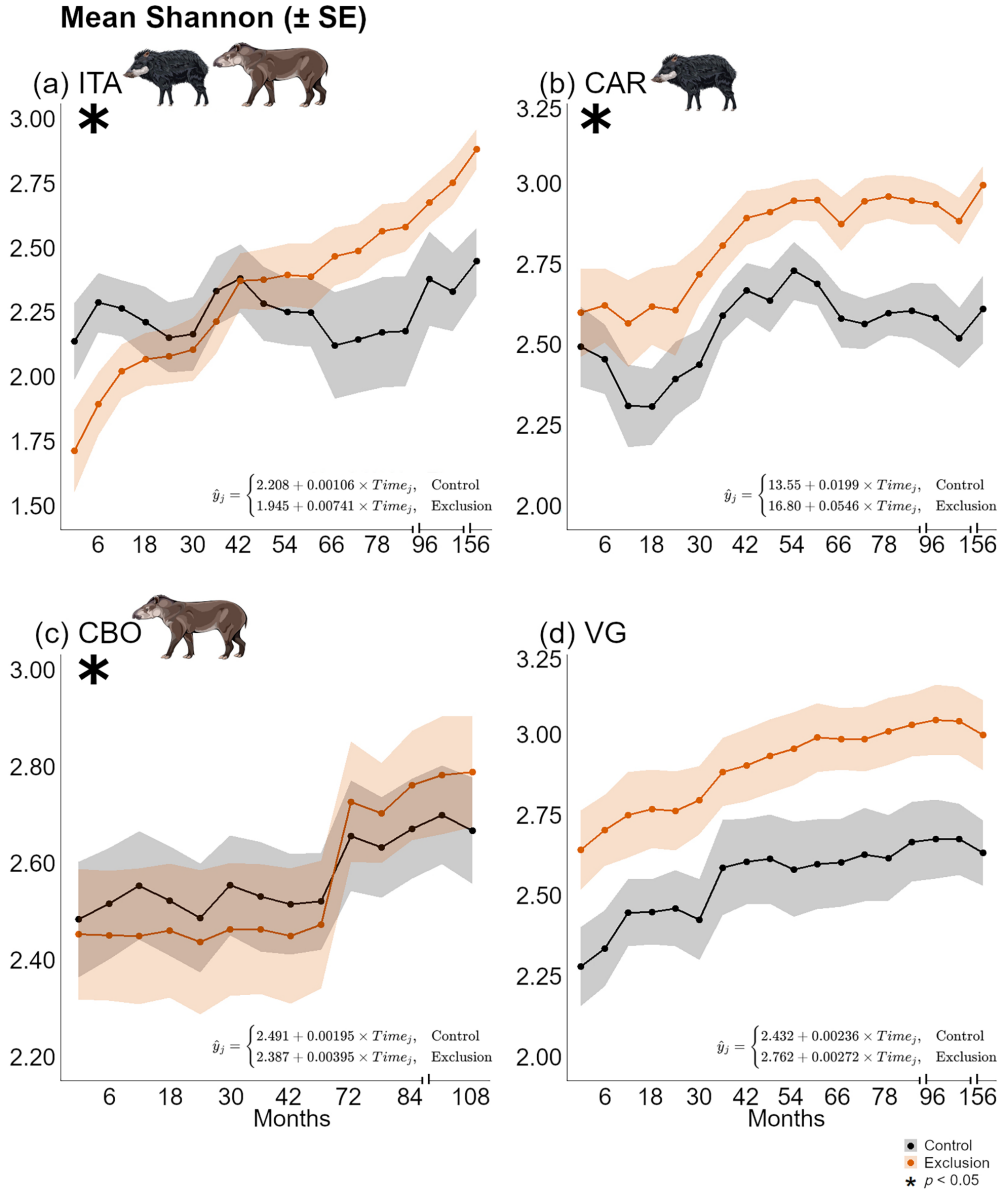
Temporal trends in Shannon diversity under control and exclusion treatments across four forest sites, based on linear mixed‐effects models. Each panel represents one site: (a) Itamambuca (ITA), (b) Ilha do Cardoso (CAR), (c) Carlos Botelho (CBO), and (d) Vargem Grande (VG). Lines represent mean Shannon diversity over time (± standard error). Linear equations represent temporal trends for each treatment, with the upper equation referring to control plots and the lower to exclusion plots. The notation *i* indicates each evaluated sampling period. Asterisks indicate statistically significant interactions (*p* < 0.05) between time and exclusion treatment, reflecting different rates of change between treatments. Tick intervals in horizontal axis represent changes in sampling constancy. Peccary and tapir illustrations by Fernanda Abra.

For Shannon diversity index (Figure 3), statistically significant treatment‐by‐time interactions were observed in ITA, CAR, and CBO, indicating that Shannon diversity increased more rapidly in the exclusion plots than in controls in those three sites. By the end of the monitoring period, mean Shannon values were higher in exclusion plots across these sites: 2.89 (SD = 0.23) in ITA versus 2.47 (SD = 0.38) in controls (Figure 3a); 3.01 (SD = 0.20) in CAR versus 2.62 (SD = 0.35) in controls (Figure 3b); and 2.80 (SD = 0.36) in CBO versus 2.68 (SD = 0.35) in controls (Figure 3c).

**FIGURE 4:**
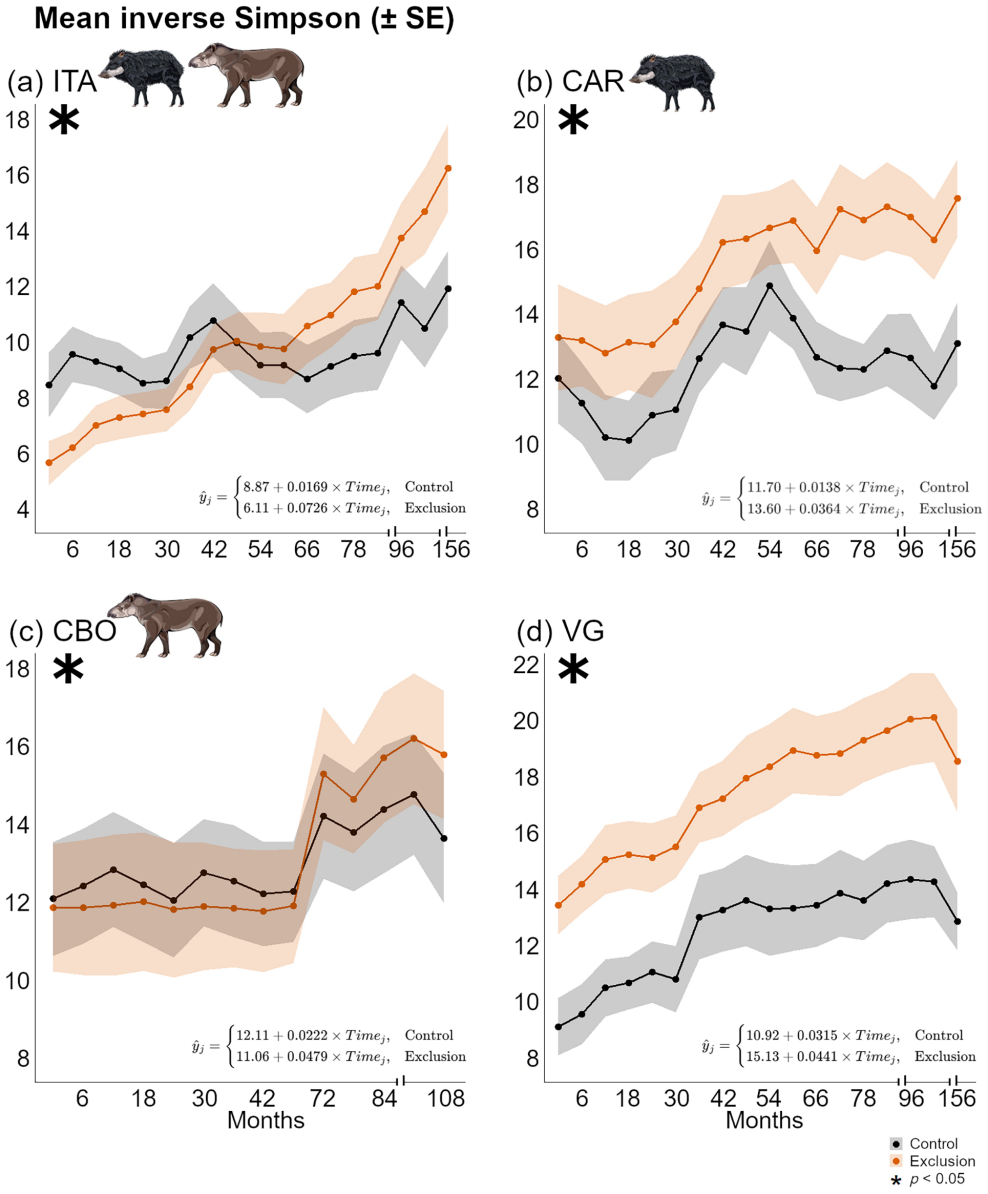
Temporal trends in inverse Simpson diversity under control and exclusion treatments across four forest sites, based on linear mixed‐effects models. Each panel represents one site: (a) Itamambuca (ITA), (b) Ilha do Cardoso (CAR), (c) Carlos Botelho (CBO), and (d) Vargem Grande (VG). Lines represent mean inverse Simpson diversity over time (± standard error). Linear equations represent temporal trends for each treatment, with the upper equation referring to control plots and the lower to exclusion plots. The notation *i* indicates each evaluated sampling period. Asterisks indicate statistically significant interactions (*p* < 0.05) between time and exclusion treatment, reflecting different rates of change between treatments. Tick intervals represent changes in sampling constancy. Peccary and tapir illustrations by Fernanda Abra.

For inverse Simpson diversity (Figure 4), all four forest sites showed significant treatment‐by‐time interactions, suggesting a widespread effect of mammal exclusion on this metric. At the final time point, mean values in exclusion plots were consistently higher: 16.41 (SD = 4.70) in ITA versus 12.07 (SD = 4.13) in controls (Figure 4a); 17.71 (SD = 3.96) in CAR versus 13.24 (SD = 4.24) (Figure 4b); 15.90 (SD = 5.21) in CBO versus 13.75 (SD = 5.29) (Figure 4c); and 18.70 (SD = 6.28) in VG versus 13.02 (SD = 3.54) (Figure 4d). Although VG exhibited consistent differences between treatments across all time points, these differences were already present at the onset of the experiment, likely reflecting baseline heterogeneity rather than treatment‐induced change. Nonetheless, exclusion still accelerated the rate of Simpson diversity increase over time in VG, although this effect was considerably weaker than in the other sites.

Both Shannon and inverse Simpson results suggest a consistent effect of exclusion on species dominance and evenness across the gradient of mammal defaunation. All these findings suggest that alpha diversity—in both richness and diversity—responded positively to mammal exclusion, particularly in forest sites with more complete mammal assemblies (e.g., ITA and CAR) as they exhibited greater rate of changes (equations in Figures 2 and 3).

### Beta diversity

**FIGURE 5:**
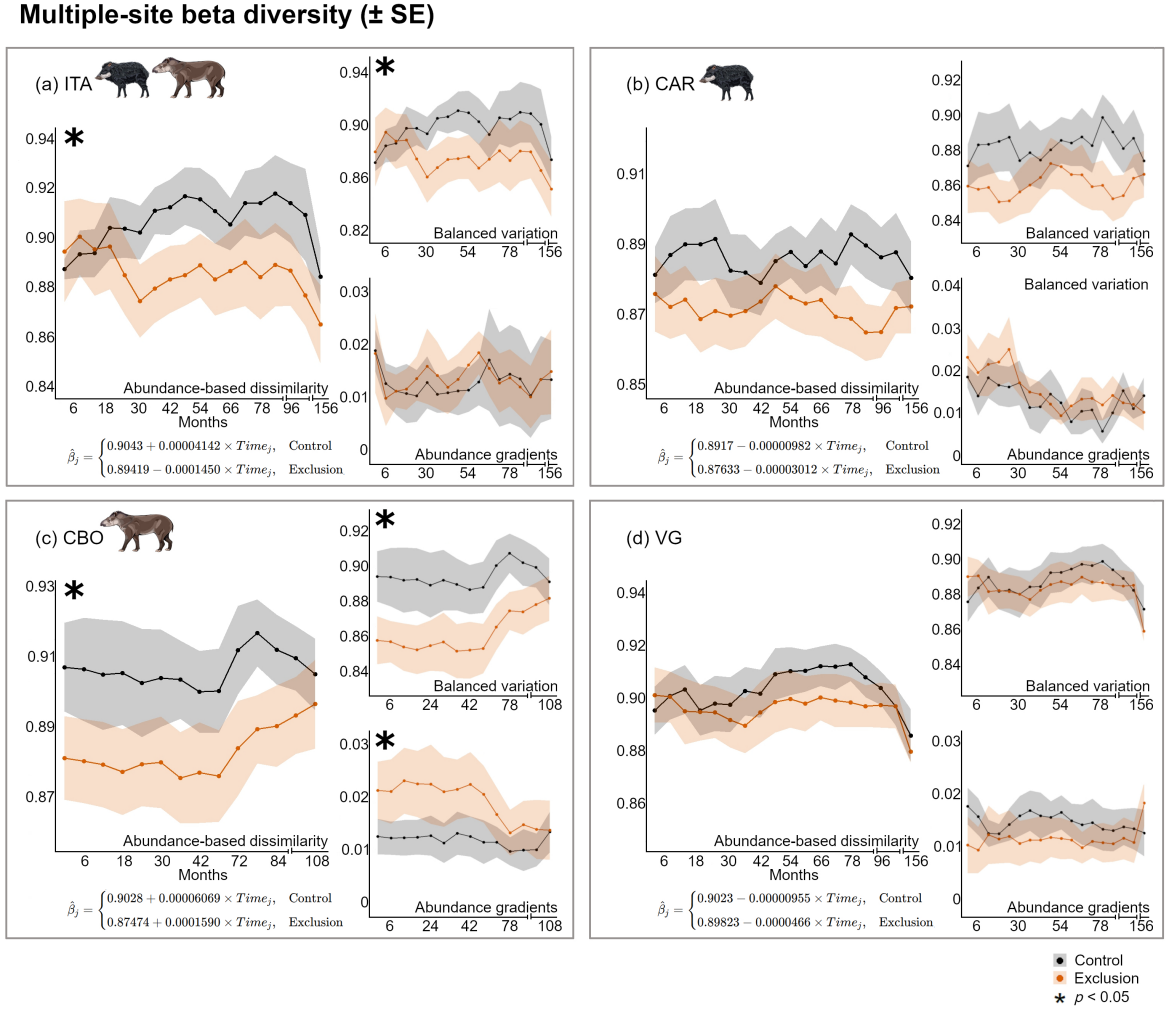
Temporal trends in multiple‐site beta diversity under control and exclusion treatments across forest sites. For each site and sampling occasion, beta diversity was calculated as Bray–Curtis multiple‐site dissimilarity among all plots within each treatment, and partitioned into its additive components: balanced variation and abundance gradients. Each panel represents one site: (a) Itamambuca (ITA), (b) Ilha do Cardoso (CAR), (c) Carlos Botelho (CBO), and (d) Vargem Grande (VG). Lines represent multiple‐site beta diversity over time (± standard error quantified by a leave‐one‐out jackknife method). Linear equations represent temporal trends for each treatment, with the upper equation referring to control plots and the lower to exclusion plots. The notation *i* indicates each evaluated sampling period. Asterisks indicate statistically significant interactions (*p* < 0.05) between time and exclusion treatment, reflecting different rates of change between treatments. Tick intervals in horizontal axis represent changes in sampling constancy. Peccary and tapir illustrations by Fernanda Abra.

In contrast to alpha diversity, beta diversity decreased over time in exclusion plots at ITA (Figure 5a), whereas it increased in control plots, revealing a clear divergence in temporal trajectories between treatments (Figure 5a). Similar patterns—declining beta diversity over time in exclusions—were also observed at CAR (Figure 5b) and VG (Figure 5d), even though the treatment‐by‐time interactions were not statistically significant. These trends suggest that seedling and sapling communities in exclusion plots became increasingly compositionally similar over time relative to control plots. By contrast, beta diversity over time increased at CBO for both treatments and with significant difference between controls and exclusion plots (Figure 5c). When beta diversity was partitioned into components representing balanced variation and abundance gradients, ITA presented significant effect for balanced variation (Appendix S2: Table S1), while CBO presented significant effect for both balanced variation and abundance gradients (Appendix S2: Table S2).

### Evaluating effects across forest sites

**FIGURE 6:**
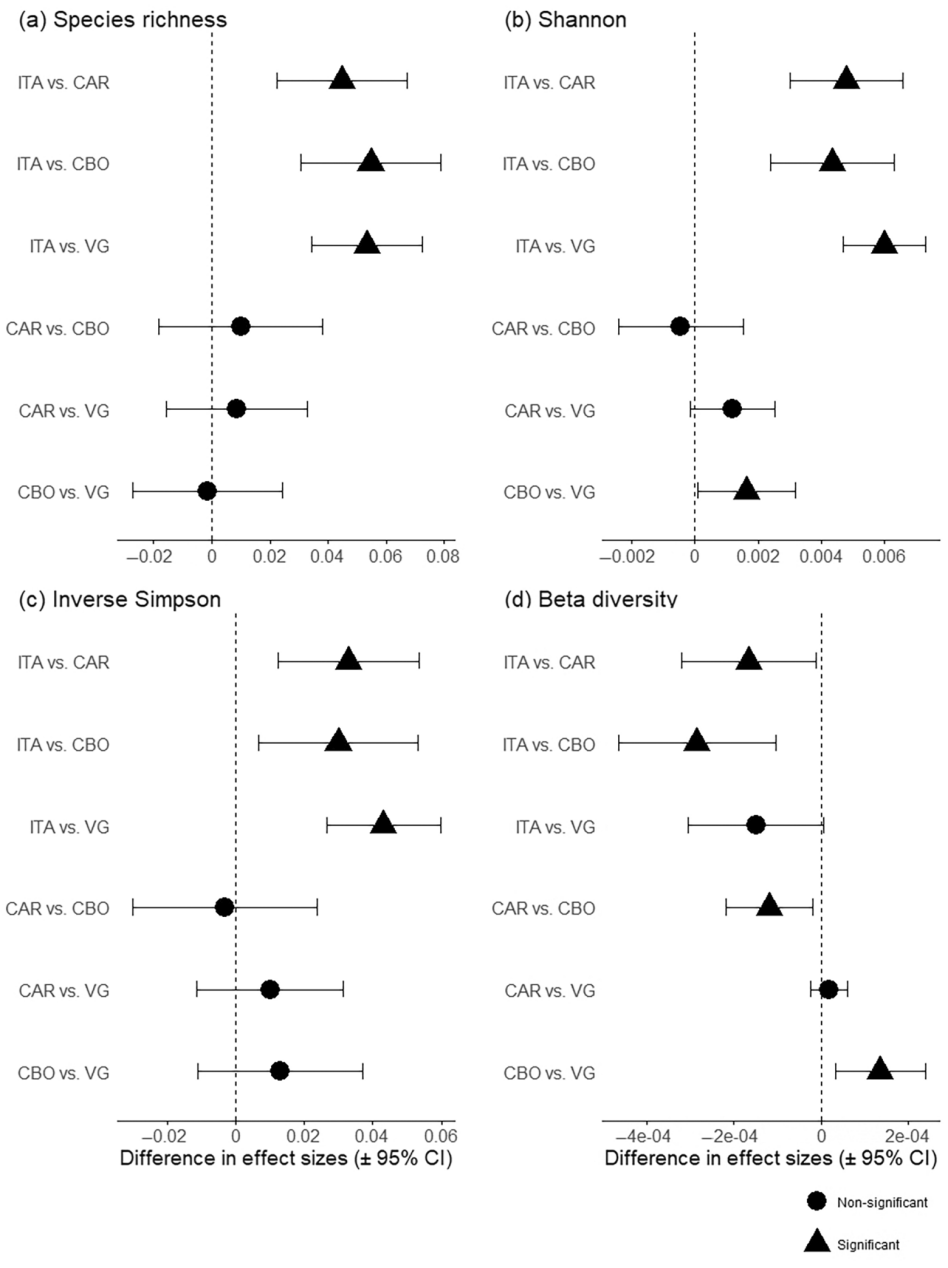
Pairwise comparisons of the time and exclusion treatment interaction across forest sites for alpha and beta diversity metrics using z‐test. Each point represents the difference between two site‐specific interaction estimates (±95% CI), calculated as (interaction in Site X) − (interaction in Site Y). Positive values indicate a less negative (or more positive) interaction in the first site of the comparison, whereas negative values indicate a more negative interaction in the first site. CIs crossing the dashed vertical line indicate non‐significant difference. Site codes: ITA, Itamambuca; CAR, Ilha do Cardoso; CBO, Carlos Botelho; VG, Vargem Grande. Each comparison is based on a single interaction estimate per site, derived from site‐specific linear models for each alpha and beta diversity metric.

Pairwise comparisons using z‐tests indicated that the effect of mammal exclusion on alpha diversity over time was significantly stronger in forest sites where exclusion more effectively reduced the influence of herbivorous mammals. ITA consistently exhibited higher positive effect sizes for all alpha diversity metrics compared to the other forest sites (Figure 6a–c). In contrast, no significant differences in effect sizes were observed among CAR, CBO, and VG, except for a higher Shannon effect size in CBO relative to VG (Figure 6b). For beta diversity, the opposite pattern was observed. ITA showed the most negative effect sizes of mammal exclusion over time, indicating a greater reduction in spatial heterogeneity compared to other sites (Figure 6d). In contrast, the difference between ITA and VG was not statistically significant, indicating that VG and ITA experienced similarly strong declines in beta diversity over time despite large differences in their mammal communities. The second forest site with higher abundance and biomass of mammals, CAR, also presented a significant difference compared to CBO. Finally, CBO—which was the only site with positive temporal trends in beta diversity for exclusion plots—showed a significantly higher (more positive) interaction estimate than VG, reflecting diversification under exclusion relative to VG.

## DISCUSSION

Our findings indicate that defaunation of large‐bodied herbivores significantly impacts seedling and sapling communities in tropical rainforests by increasing alpha diversity over time while simultaneously promoting taxonomic biotic homogenization. As hypothesized, the effect of exclusion was most pronounced in forest sites with more complete mammal assemblies (Appendix S1: Table S1), leading to substantial divergence between exclusion and control plots in these forests. Yet, in our long‐term experiment, we ascertained opposite effects of defaunation on alpha and beta diversity of tropical rainforests, an unexpected and unreported result.

### Alpha diversity

Contrary to our initial expectation that a lack of herbivory, trampling and disturbance by medium to large‐bodied herbivores would lead to increased competitive dominance of a few selected plant species, resulting in decreased alpha diversity (Koerner et al., 2018), we observed an increase in alpha diversity with defaunation. These findings are consistent with other studies in seasonal Atlantic Forest (e.g., Villar & Medici, 2021) that also question traditional models derived from grassland ecosystems predicting increased local (alpha) plant diversity with increased herbivory in tropical forests. In our experiment, the reduction in herbivory pressure allowed a broader range of species to establish and thrive, particularly in the forest site with a more complete mammal assembly, where we observed significant increases in species richness, Shannon, and inverse Simpson indices with exclusion. However, the effects were less pronounced at other forest sites, suggesting that localized factors, such as differences in mammal community composition and herbivore abundance, play critical roles in shaping local diversity dynamics (Villar & Medici, 2021).

This unexpected outcome is consistent with the reduction in top‐down regulation by seed, seedling, and sapling predators, which have been shown to suppress rare species and thus reduce alpha diversity in tropical forests when present (Galetti et al., 2021; Villar et al., 2022; Villar & Medici, 2021). A previous study from our long‐term experiment demonstrated that on forests where voracious large seed, seedling, and sapling predators (such as white‐lipped peccaries) are abundant, these herbivores reduce seedling recruitment by 60% and, simultaneously, reduce the share and diversity of rare species in plant communities in benefit of greater evenness among dominant species (Keuroghlian & Eaton, 2009; Villar & Medici, 2021). Our results are consistent with these findings, as our meta‐analytical approach clearly showed that stronger positive effects of exclusion on alpha diversity were found in areas where white‐lipped peccaries were present. It is likely that the strong reduction in recruitment caused by these voracious and abundant seed, seedling, and sapling predators creates a strong filter against the arrival and recruitment of rare species, constraining community size in a “lottery” fashion so that only sufficiently common species can recruit in plots.

This finding underscores the intricate interplay between herbivory and plant community assembly in tropical forests, where large herbivores may facilitate shifts in dominance that benefit some species at the expense of others (Kurten et al., 2015; Villar & Medici, 2021). In addition, it has been hypothesized that these interactions might be further complicated by the presence of arboreal and small‐sized mammals, birds, bats, and other animals with free access to the plots, such as primates, which are thought to influence plant recruitment and diversity when released from the competitive pressure of larger herbivores (Bueno et al., 2013; Kurten & Carson, 2015). Yet, in our study, we did not find an effect of seed mesopredators, neither of tapirs nor of collared peccaries, on alpha diversity in areas where white‐lipped peccaries were absent (CBO, VG). Instead, our results clearly suggest that the voracious and herd‐forming white‐lipped peccary is a keystone species exhorting a strong filtering effect on rare plant species with knock‐on effects on the community assembly and alpha diversity of tropical forests.

While the apparent increase in alpha diversity following the loss of white‐lipped peccaries may suggest a positive outcome, this pattern likely reflects a short‐term relaxation of top‐down control and ecological filtering, rather than a genuine improvement in forest diversity. The absence of these large herbivores allows a subset of dominant species—such as *Euterpe edulis* and *Merostachys neesii—*to proliferate, thereby suppressing recruitment of subordinate species and reducing spatial turnover. For instance, previous findings have shown that the hyperdominance aspect of *Euterpe edulis* leads plant growth forms to struggle and compete more upon release from large herbivores, thereby disrupting plant interaction dynamics (Souza et al., 2022). In the long term, this trend can compromise ecosystem resilience, lower functional diversity, and homogenize plant communities. Therefore, forests lacking white‐lipped peccaries and where arboreal species are still abundant may experience a transient increase in alpha diversity, but at the cost of biotic homogenization and loss of ecological complexity, particularly in plant–mammal interaction networks.

### Beta diversity

In contrast to alpha diversity, our hypothesis regarding taxonomic biotic homogenization was supported: beta diversity decreased over time with defaunation. In the forest site with more complete assemblies of medium and large herbivores (ITA), their exclusion led to more similar plant communities across the forest. Closer inspection of these results revealed that the decline of beta diversity was due to reduced spatial turnover in species composition, and a concomitant increase in the relative abundance of the most dominant plant species at the landscape scale in exclusion plots in these forests, such as the hyperdominant keystone palm *Euterpe edulis*, the bamboo *Merostachys* cf. *neesii*, and the fern *Polybotrya cylindrica*. This homogenization effect was also observed, though to a lesser extent, at the site where the white‐lipped peccary was present but not the tapir (CAR), but not in the other forest sites. Again, this trend points to the keystone effect of seed, seedling, and sapling predation by white‐lipped peccaries as a facilitator of increases in spatial heterogeneity in plant community composition.

Although the balanced variation component of beta diversity was statistically significant for ITA, its trajectory closely mirrored that of overall beta diversity, indicating that most of the observed homogenization arose from shifts in the *relative abundances* of species among plots rather than from abundance gradients. Abundance gradients contributed minimally to total beta diversity, suggesting that changes in overall abundance played only a negligible role in the observed patterns.

Secondary factors, such as reproductive events and changes in seasonality due to global warming, may also have boosted the increase in the abundance of these palm and bamboo species. Bamboo species experienced a reproduction event in 2019 after 10 years of experiment start, which contributed to an increase in the abundance of seedlings following reproduction and dispersal (Souza et al., 2022). Their ability to persist under disturbed situations, such as defaunation, along with vegetative propagation and regeneration, might contribute to *Merostachys neesii* success in dispersing and establishing better in exclosure plots (Buckingham et al., 2011; Calderón‐Sanou et al., 2019; Rother et al., 2013). On the other hand, a previous study has shown that *Euterpe edulis* increases exponentially once released from large herbivore impacts and maintains its abundance higher compared to open plots even when time passes (Souza et al., 2022). The phenology, dispersal, and germination dynamics of palm species are suggested to rely strongly on climate and seasonality (Matos & Watkinson, 2006; Muscarella et al., 2020; Sales et al., 2021). Therefore, we argue that the long‐term response of this palm species to defaunation in our experiment may be tied to and buffered by variation in climate conditions.

However, an alternative explanation is also possible. Rather than mammal exclusion actively causing homogenization, it may have prevented the natural increase in heterogeneity that would have occurred in control plots over time due to ecological succession or unregulated herbivory. In this view, exclusion could be interpreted as slowing down heterogenization, particularly in sites with greater mammal biomass. We acknowledge this possibility and highlight that our interpretation of biotic homogenization is always made in comparison to the control treatment at each site. Moreover, all forest sites share broadly similar biogeographic and successional histories, reinforcing the interpretation that differences in beta diversity trajectories arise from variation in mammal community composition and the intensity of defaunation.

Results show that, in addition to precluding the recruitment of rare species (see previous paragraphs), the predatory and disturbance impact of white‐lipped peccaries also appears to limit the recruitment of the most dominant plant species at the forest level, in benefit of subdominant species. Previous studies demonstrate that large mammals like peccaries and tapirs in the Atlantic Forest are generalist consumers attracted to hotspots of hyperdominant plants that produce large amounts of fruits, such as the palm *Euterpe edulis* (Villar et al., 2022). Other studies have also demonstrated the significant role white‐lipped peccaries play in controlling populations of the palm *Euterpe edulis* through seed predation, which may reduce the species' dominance (Keuroghlian & Eaton, 2009). This behavior has strong effects on the spatial distribution of seedling communities (Valverde et al., 2021), and on spatial gradients in plant biomass, productivity, and nutrient cycling in these forests (Villar et al., 2021, 2022; Villar & Medici, 2021). Both recruitment limitation of the most dominant species and the creation of spatially heterogeneous gradients and disturbances by these large predatory seed and herbivores can promote spatial heterogeneity and subsequently increase beta diversity. Additionally, it is possible that seed dispersal by tapirs might have contributed to generate higher beta diversity in open plots of the forest site with the most complete mammal assembly (e.g., compare results from ITA vs. CAR forests).

Yet, results from the forest site where tapirs but not white‐lipped peccaries were present (CBO) revealed a contrasting pattern. Instead of a decrease, beta diversity increased in exclusion plots over time, indicating that the removal of tapirs—or any other remaining medium‐sized mammals—did not generate the homogenization observed in sites with peccaries. Also, both balanced variation and abundance gradients were statistically significant in CBO; however, abundance gradients contributed only a minimal fraction of overall beta diversity and likely represent a weak or artifactual signal. The increase in beta diversity was therefore driven primarily by balanced variation, reflecting reductions in the dominance of a few previously abundant species and greater evenness among subdominant taxa. Moreover, CBO harbors a rich assemblage of arboreal frugivores, particularly primates (Appendix S1), whose seed dispersal combined with natural regeneration may have enhanced spatial heterogeneity in exclusion plots despite the low biomass of terrestrial herbivores. This pattern suggests that the contribution of other seed dispersers to spatial biotic heterogeneity becomes more apparent in forests lacking the strong disturbance regime imposed by white‐lipped peccaries.

### Synergic effects of large herbivores

Overall, the spatial biotic homogenization observed in exclusion plots at the forest site where the exclusion treatment removed the most complex mammals' community (ITA) indicates that the loss of these large‐bodied herbivores can lead to more spatially uniform and potentially less resilient ecosystems, with significant implications for conservation and ecosystem management strategies. These findings align with recent studies highlighting the role of large herbivores in maintaining landscape‐scale heterogeneity by modulating competitive interactions among plant species (e.g., Ribeiro et al., 2025; Trepel et al., 2024). While the effects of white‐lipped peccary appear critical, the interaction between different keystone herbivore species simultaneously regulating plant communities appears to have additional effects in shaping plant community dynamics and assembly over time. While defaunation of white‐lipped peccaries alone had moderate effects on alpha and beta diversity, and defaunation of tapirs alone had no effect, the exclusion of both herbivores at the same forest resulted in substantially higher impacts on all metrics.

It is possible that long‐term differential defaunation of sites has already impacted plant community assembly and composition in these and other areas of the Atlantic Forest. For example, total seedling and sapling species richness across all plots in each forest was lowest at ITA (170 seedling and sapling taxa), the forest with the most complete mammal assembly, where our experiment shows large herbivores caused a stronger decline in species richness. Species richness was higher in CAR (183), then CBO (186), and highest in the most defaunated site, VG (229). This gradient in whole site species richness matches the predicted long‐term effects of the demise of the filtering effect of these keystone mammalian species on (rare) plant species richness ascertained in our experiment. Additionally, the abundance of adult hyperdominant palms *Euterpe edulis* and its recruitment is almost twice at VG, the most defaunated site, than at ITA (Villar et al., 2022; Villar & Medici, 2021), despite the proximity of these sites. These patterns are consistent with the long‐term impact of differential defaunation gradients on alpha diversity and plant dominance as predicted by the results of our experiment, namely that defaunation of keystone white‐lipped peccaries and tapirs would lead to a forest‐wide increase in (rare) species richness but also to a concomitant increase of hyperdominant species.

## CONCLUSIONS

Our findings underscore the complex interplay between herbivores and plant community assembly, where large‐bodied mammals may facilitate shifts in dominance that benefit some species at the expense of others (Villar & Medici, 2021). Furthermore, we propose that the synergistic interactions between keystone herbivores, such as the combined effects of white‐lipped peccaries and tapirs, play a critical role in regulating plant community structure and diversity at both local and landscape scales. These interactions may enhance spatial insurance and compositional turnover, contributing to the overall resilience of tropical forest ecosystems.

Collectively, our results indicate that large‐bodied herbivores in tropical rainforests cause a strong increase in alpha diversity, potentially by limiting community size and filtering rare species, but simultaneously preventing hyperdominant species to thrive, allowing subdominant species to “climb up” the dominance hierarchy. Yet these shifts in dominance are not enough to compensate for the strong filtering of rare species, thus yielding outcomes of herbivory different from those reported in many grassland ecosystems (Abella et al., 2019; Milligan et al., 2016; Speed et al., 2013; Watts et al., 2019). On the other hand, these processes also contribute to promoting a concomitant increase in spatial heterogeneity in species composition, leading to increases in beta diversity in areas with conserved large herbivore assemblies. Given these results, we speculate that large mammalian herbivores might contribute substantially to the observed high levels of beta diversity observed in Neotropical forests reported elsewhere (Condit et al., 2002; Myers et al., 2013). Yet, our experimental results clearly challenge the speculative hypothesis that (seed, seedling and saplings) predation by ground‐dwelling large‐bodied herbivores results in high levels of alpha diversity respective to beta diversity (Terborgh, 2015), as evidence from our long‐term experiment points in the opposite direction.

Forest ecosystems worldwide are currently facing unprecedented challenges due to multiple anthropogenic pressures, such as defaunation, habitat fragmentation, and climate change, which are widespread and ongoing (Benítez‐López et al., 2019; Bogoni et al., 2020; Galetti et al., 2021). These pressures are driving rapid changes in the composition and structure of these ecosystems (Dornelas et al., 2014), leading to significant shifts in biodiversity at multiple scales (Dornelas et al., 2014; Ripple et al., 2020). Therefore, it is essential to understand the roles of large‐bodied wild herbivores in maintaining biodiversity across different scales, including the specific impacts of white‐lipped peccaries and tapirs on plant community diversity. Our study provides detailed insights into the effects of defaunation on seedling and sapling communities in tropical rainforests. The observed spatial forest‐wide biotic homogenization caused by defaunation suggests that the loss of large herbivore species could lead to more uniform, less heterogeneous, and potentially less resilient tropical rainforests. This has important implications for conservation strategies, underscoring the need to preserve herbivorous mammal populations to maintain biodiversity, ecosystem functionality, and its associated ecological services.

## AUTHOR CONTRIBUTIONS

Sergio Nazareth, Carlos R. Brocardo, Luana Hortenci, and Gabriela Schmaedecke installed the plot experiment, monitored seedling and sapling specimens, and collected data during the early years of the experiment. Valesca Zipparro, Sérgio Nazareth, and Mauro Galetti designed, set up, and maintained the experiment, monitored seedling specimens, and collected data. Luiz G. S. Ribas wrote the first draft, performed data analysis, and prepared the figures. Valesca Zipparro, Mauro Galetti, Yuri Souza, Carlos R. Brocardo, Luana Hortenci, Gabriela Schmaedecke, and Nacho Villar contributed substantially to revisions and final writing.

## CONFLICT OF INTEREST STATEMENT

The authors declare no conflicts of interest.

## Supporting information


Appendix S1.

Appendix S2.

## Data Availability

Data and code (LABIC‐UNESP, 2026) are available in Zenodo at https://doi.org/10.5281/zenodo.18261122.
