# Supplementary material for: Mammal defaunation leads to biotic homogenization of plant communities in tropical rainforests
Source: Ecology. 2026 Mar 16;107(3):e70341. doi: 10.1002/ecy.70341 (PMC12991963; doi:10.1002/ecy.70341)
Supplement: Supplementary file 1 — Appendix S1. [file ECY-107-e70341-s001.pdf]

Appendix S1

Mammal defaunation leads to biotic homogenization of plant communities in tropical rainforests

Luiz Guilherme dos Santos Ribas, Nacho Villar, Valesca Zipparro, Sérgio Nazareth, Yuri Souza, Carlos Rodrigo Brocardo, Gabriela Schmaedecke, Luana Hortenci, Rafael Souza Cruz Alves, Mauro Galetti

Ecology

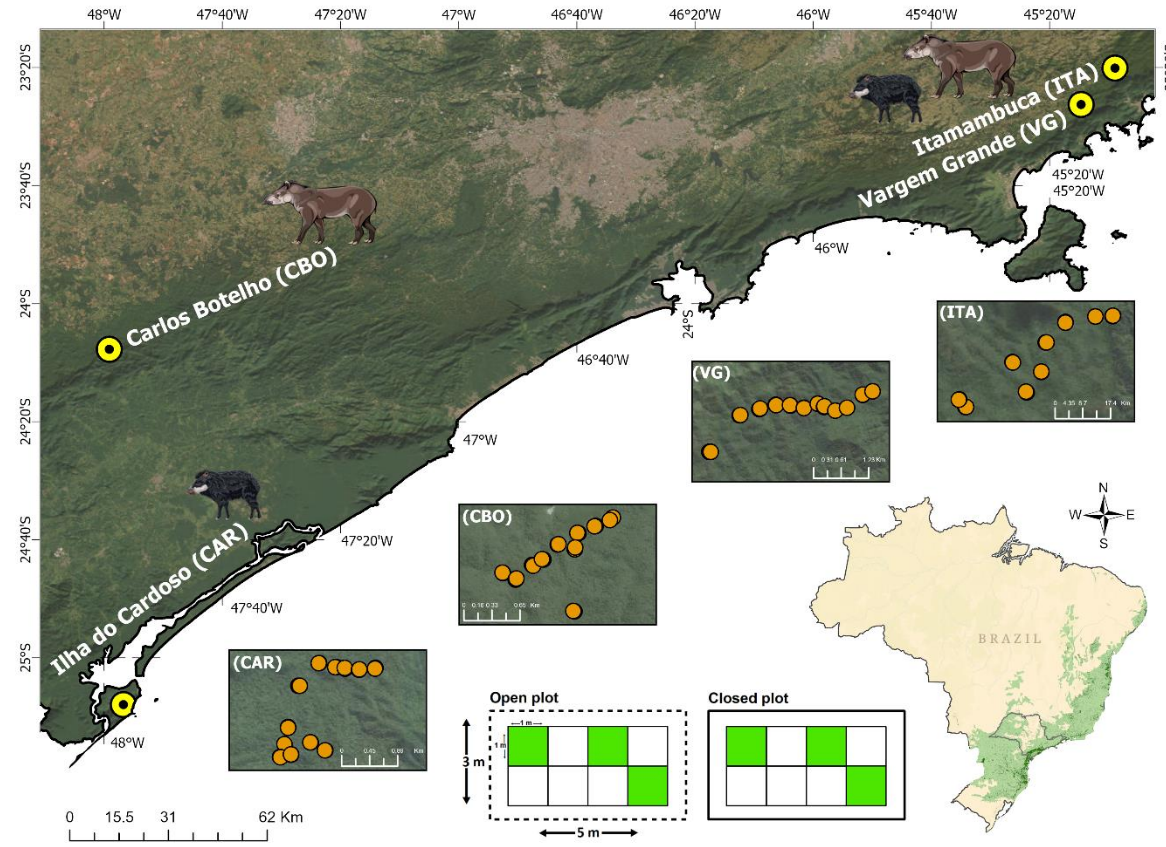

Figure S1. Location of the The DEFAU-Biota project experimental areas within the Atlantic Forest of São Paulo State, Brazil. The map shows the spatial distribution of the four forest sites, each characterized by a distinct combination of large mammals: Itamambuca (ITA), where white-lipped peccaries (Tayassu pecari) and tapirs (Tapirus terrestris) are present; Ilha do Cardoso (CAR), where only peccaries are present; Carlos Botelho (CBO), where only tapirs are present; and Vargem Grande (VG), where both species are absent. The diagram also illustrates the spatial layout of paired experimental plots at each site, including control plots (open) and exclusion plots (closed). Peccary and tapir illustrations by Fernanda Abra.

Mammal Abundance data

1-Carlos Botelho State Park (CBO):

CBO harbors a diverse assemblage of non-volant mammals, with more than 50 species recorded (Brocardo et al. 2012). This site supports a high density of primates; however, the densities of ungulates and large rodents—commonly targeted as game species—are notably low(Galetti et al. 2017). Line transect censuses estimated a relative abundance of 0.04 individuals/10 km for tapirs and 0.02 individuals/10 km for agoutis, with no records of peccaries or deer detected using this method (Table S1).

2- Itamambuca (ITA):

Rocha-Mendes et al. (2015) reported 58 non-volant mammal species in the Núcleo Santa Virgínia of Serra do Mar State Park, which includes both ITA and VG. In ITA, line transect censuses revealed relatively high ungulate abundance compared to CBO and VG, with white-lipped peccaries recorded at 0.13 individuals/10km (Table S1).

3- Vargem Grande (VG):

VG exhibited the lowest abundance of ground-dwelling large mammals among the study sites (Table S1). No ungulate records were obtained through line transect surveys.

4-Ilha do Cardoso State Park (CAR):

This island supports 70 mammal species, although it lacks both the largest Neotropical ungulate (the lowland tapir) and the top predator (the jaguar) (Estado de São Paulo 2001). Nevertheless, CAR showed the highest abundance of large-seed predators among the study sites, with agoutis recorded at 0.49 individuals/10 km, white-lipped peccaries at 0.28, collared peccaries at 0.02, and deer also at 0.02 (Table S1).

Table S1. Relative abundance (individuals/10 km) of medium- and large-bodied mammals at our study sites, estimated through line transect censuses (for methodological details, see Galetti et al. 2017). NP = not present at the site; NR = not recorded, but potentially present.

Species	CAR	CBO	ITA	VG
Alouatta guariba	1.01	0.25	0.11	NR
Brachyteles arachnoides	NP	0.72	0.04	NR
Callithrix aurita	NP	NP	0.51	0.72
Sapajus nigritus	NP	0.61	0.17	0.16
**Primates**	1.01	1.58	0.83	0.87
Cerdocyon thous	0.02	NR	NR	NR
Eira barbara	0.02	0.02	NR	0.08
Nasua nasua	0.23	0.09	0.04	0.08
**Small carnivores**	0.26	0.11	0.04	0.16
Dasyprocta sp	0.49	0.02	NR	0.08
Hydrochoerus hydrochaeris	NR	NR	NR	0.08
**Large rodents**	0.49	0.02	NR	0.16
Mazama sp	0.02	NR	NR	NR
Dicotyles tajacu	0.02	NR	NR	NR
Tayassu pecari	0.28	NR	0.13	NR
Tapirus terrestris	NP	0.04	NR	NR
**Ungulates**	0.31	0.04	0.13	NR

References

Brocardo, C. R., R. Rodarte, R. da S. Bueno, L. Culot, and M. Galetti. 2012. Mamíferos não voadores do Parque Estadual Carlos Botelho, continuum florestal do Paranapiacaba. Biota Neotropica:198–208.

Estado de São Paulo. 2001. Plano de Manejo do Parque Estadual da Ilha do Cardoso. São Paulo.

Galetti, M., C. Brocardo, R. Begotti, L. Hortenci, F. Rocha-Mendes, C. Bernardo, R. Bueno, R. Nobre, R. Bovendorp, and R. Marques. 2017. Defaunation and biomass collapse of mammals in the largest Atlantic forest remnant. Animal Conservation 20:270–281.

Rocha-Mendes, F., C. L. Neves, R. de A. Nobre, R. M. Marques, G. V. Bianconi, and M. Galetti. 2015. Non-volant mammals from Núcleo Santa Virgínia, Serra do Mar State Park, São Paulo, Brazil. Biota Neotropica 15:e20140008.
