# Supplementary material for: Mammal defaunation leads to biotic homogenization of plant communities in tropical rainforests
Source: Ecology. 2026 Mar 16;107(3):e70341. doi: 10.1002/ecy.70341 (PMC12991963; doi:10.1002/ecy.70341)
Supplement: Supplementary file 2 — Appendix S2. [file ECY-107-e70341-s002.pdf]

Appendix S2

Mammal defaunation leads to biotic homogenization of plant communities in tropical rainforests

Luiz Guilherme dos Santos Ribas, Nacho Villar, Valesca Zipparro, Sérgio Nazareth, Yuri Souza, Carlos Rodrigo Brocardo, Gabriela Schmaedecke, Luana Hortenci, Rafael Souza Cruz Alves, Mauro Galetti

*Ecology*

Table S1. Temporal trends in beta diversity partitioned into balanced variation for Itamambuca (ITA). Results are interpreted because overall beta diversity showed a statistically significant effect for this site.

| Term | Estimate | Std. Error | t value | P value | ± 95% CI |
|---|---|---|---|---|---|
| Intercept | 8.91E-01 | 4.01E-03 | 222.203 | <2e-16 | [0.8828891, 0.8992257] |
| Time | 3.87E-05 | 5.95E-05 | 0.651 | 0.52 | [-0.00008249502, 0.0001599082] |
| Exclusion treatment | -1.12E-02 | 5.67E-03 | -1.966 | 0.058 | [-0.02270343, 0.0004000245] |
| Time and exclusion treatment interaction | -1.84E-04 | 8.42E-05 | -2.19 | 0.036 | [-0.0003556619, -0.00001285206] |

Table S2. Temporal trends in beta diversity partitioned into balanced variation for lha do Cardoso State Park (CAR). Results are not interpreted because overall beta diversity showed no statistically significant effect for this site.

| Term | Estimate | Std. Error | t value | P value | ± 95% CI |
|---|---|---|---|---|---|
| Intercept | 8.77E-01 | 2.22E-03 | 394.94 | < 2e-16 | [0.8722777, 0.8813221] |
| Time | 2.07E-05 | 3.29E-05 | 0.627 | 0.535 | [-0.00004643722, 0.00008776277] |
| Exclusion treatment | -1.92E-02 | 3.14E-03 | -6.1 | 8.13E-07 | [-0.02554714, -0.01275653] |
| Time and exclusion treatment interaction | 1.62E-05 | 4.66E-05 | 0.347 | 0.731 | [-0.00007872894, 0.0001110585] |

Table S3. Temporal trends in beta diversity partitioned into balanced variation for Carlos Botelho State Park (CBO). Results are interpreted because overall beta diversity showed a statistically significant effect for this site.

| Term | Estimate | Std. Error | t value | P value | ± 95% CI |
|---|---|---|---|---|---|
| Intercept | 8.90E-01 | 2.36E-03 | 377.921 | < 2e-16 | [0.8853087, 0.8950314255] |
| Time | 7.52E-05 | 4.09E-05 | 1.84 | 0.07809 | [-0.000009126019, 0.0001594919] |
| Exclusion treatment | -3.85E-02 | 3.33E-03 | -11.558 | 2.71E-11 | [-0.04537636, -0.0316262855] |
| Time and exclusion treatment interaction | 1.75E-04 | 5.78E-05 | 3.024 | 0.00587 | [0.00005544961, 0.0002939113] |

Table S4. Temporal trends in beta diversity partitioned into balanced variation for Vargem Grande (VG). Results are not interpreted because overall beta diversity showed no statistically significant effect for this site.

| Term | Estimate | Std. Error | t value | P value | ± 95% CI |
|---|---|---|---|---|---|
| Intercept | 8.87E-01 | 2.43E-03 | 365.789 | <2e-16 | [0.8821508, 0.8920305] |
| Time | 8.06E-06 | 3.60E-05 | 0.224 | 0.824 | [-0.00006524069, 0.00008135464] |
| Exclusion treatment | 1.22E-03 | 3.43E-03 | 0.355 | 0.725 | [-0.005769823, 0.008202181] |
| Time and exclusion treatment interaction | -7.96E-05 | 5.09E-05 | -1.565 | 0.128 | [-0.0001832798, 0.00002403733] |

Table S5. Temporal trends in beta diversity partitioned into abundance gradients for Itamambuca (ITA). Results are interpreted because overall beta diversity showed a statistically significant effect for this site.

| Term | Estimate | Std. Error | t value | P value | ± 95% CI |
|---|---|---|---|---|---|
| Intercept | 1.33E-02 | 9.48E-04 | 14.016 | 3.30E-15 | [0.01134951, 0.01520943] |
| Time | 2.71E-06 | 1.41E-05 | 0.193 | 0.848 | [-0.00002592376, 0.00003134981] |
| Exclusion treatment | 1.04E-03 | 1.34E-03 | 0.776 | 0.444 | [-0.001689876, 0.003768869] |
| Time and exclusion treatment interaction | -2.18E-06 | 1.99E-05 | -0.11 | 0.913 | [-0.00004267982, 0.00003831723] |

Table S6. Temporal trends in beta diversity partitioned into abundance gradients for lha do Cardoso State Park (CAR). Results are not interpreted because overall beta diversity showed no statistically significant effect for this site.

| Term | Estimate | Std. Error | t value | P value | ± 95% CI |
|---|---|---|---|---|---|
| Intercept | 1.49E-02 | 9.89E-04 | 15.021 | 4.78E-16 | [0.0128445, 0.01687434] |
| Time | -3.05E-05 | 1.47E-05 | -2.076 | 0.046 | [-0.00006037877, -0.0000005801551] |
| Exclusion treatment | 3.78E-03 | 1.40E-03 | 2.704 | 0.0109 | [0.0009339096, 0.006633316] |
| Time and exclusion treatment interaction | -3.65E-05 | 2.08E-05 | -1.757 | 0.0885 | [-0.00007875196, 0.000005816041] |

| Term | Estimate | Std. Error | t value | P value | ± 95% CI |
|---|---|---|---|---|---|
| Intercept | 1.26E-02 | 6.01E-04 | 21.002 | < 2e-16 | [0.01137744, 0.01385728] |
| Time | -1.45E-05 | 1.04E-05 | -1.391 | 0.177 | [-0.00003599778, 0.000007009114] |
| Exclusion treatment | 1.05E-02 | 8.50E-04 | 12.294 | 7.56E-12 | [0.008691791, 0.01219882] |
| Time and exclusion treatment interaction | -7.64E-05 | 1.47E-05 | -5.184 | 2.61E-05 | [-0.0001067930, -0.00004597202] |

Table S7. Temporal trends in beta diversity partitioned into abundance gradients for Carlos Botelho State Park (CBO). Results are interpreted because overall beta diversity showed a statistically significant effect for this site.

| Term | Estimate | Std. Error | t value | P value | ± 95% CI |
|---|---|---|---|---|---|
| Intercept | 1.52E-02 | 5.68E-04 | 26.802 | < 2e-16 | [0.01406867, 0.01638297] |
| Time | -1.76E-05 | 8.43E-06 | -2.088 | 0.04481 | [-0.00003477308, -0.000000433314 |
| Exclusion treatment | -5.28E-03 | 8.03E-04 | -6.577 | 2.07E-07 | [-0.006920211, -0.003647287] |
| Time and exclusion treatment interaction | 4.26E-05 | 1.19E-05 | 3.572 | 0.00114 | [0.00001830477, 0.00006686853] |

Table S8. Temporal trends in beta diversity partitioned into abundance gradients for Vargem Grande (VG). Results are not interpreted because overall beta diversity showed no statistically significant effect for this site.
